# Nutrient and Stress Sensing in Pathogenic Yeasts

**DOI:** 10.3389/fmicb.2019.00442

**Published:** 2019-03-08

**Authors:** Julian C. Rutherford, Yong-Sun Bahn, Bert van den Berg, Joseph Heitman, Chaoyang Xue

**Affiliations:** ^1^Institute for Cell and Molecular Biosciences, Newcastle University, Newcastle upon Tyne, United Kingdom; ^2^Department of Biotechnology, Yonsei University, Seoul, South Korea; ^3^Department of Molecular Genetics and Microbiology, Duke University Medical Center, Durham, NC, United States; ^4^Public Health Research Institute, Rutgers University, Newark, NJ, United States; ^5^Department of Molecular Genetics, Biochemistry and Microbiology, Rutgers University, Newark, NJ, United States

**Keywords:** yeast, nutrient sensing, transceptor, G protein-coupled receptor, Mep2, fungal pathogen, stress response, Tor

## Abstract

More than 1.5 million fungal species are estimated to live in vastly different environmental niches. Despite each unique host environment, fungal cells sense certain fundamentally conserved elements, such as nutrients, pheromones and stress, for adaptation to their niches. Sensing these extracellular signals is critical for pathogens to adapt to the hostile host environment and cause disease. Hence, dissecting the complex extracellular signal-sensing mechanisms that aid in this is pivotal and may facilitate the development of new therapeutic approaches to control fungal infections. In this review, we summarize the current knowledge on how two important pathogenic yeasts, *Candida albicans* and *Cryptococcus neoformans*, sense nutrient availability, such as carbon sources, amino acids, and ammonium, and different stress signals to regulate their morphogenesis and pathogenicity in comparison with the non-pathogenic model yeast *Saccharomyces cerevisiae*. The molecular interactions between extracellular signals and their respective sensory systems are described in detail. The potential implication of analyzing nutrient and stress-sensing systems in antifungal drug development is also discussed.

## Carbon Sensing in Fungal Pathogenesis

**FIGURE 1:**
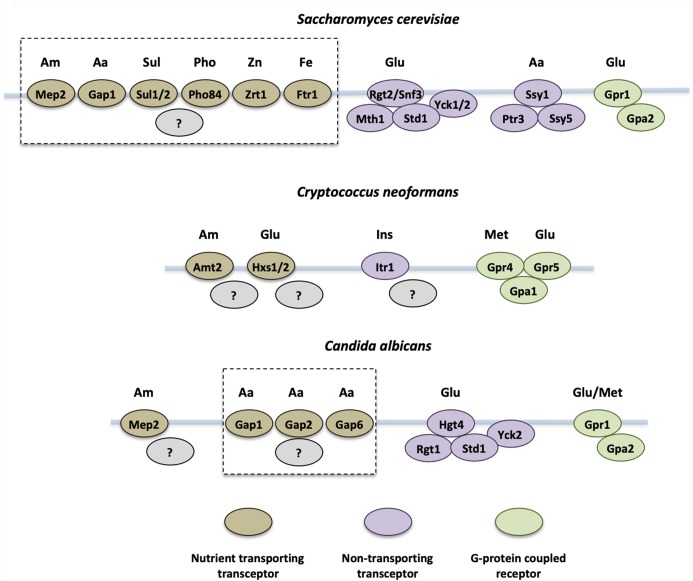
Yeast nutrient receptors that sense external nutrient availability. Nutrient sensing transceptors and G-protein coupled receptors in *Saccharomyces cerevisiae, Cryptococcus neoformans*, and *Candida albicans* are shown. Nutrient sensors for glucose (Glu), amino acids (Aa), ammonium (Am), sulfate (Sul), phosphate (Pho), zinc (Zn), iron (Fe), methionine (Met), and inositol (Ins) have been identified. Dashed lines group those transceptors that rapidly activate the PKA pathway by an unknown mechanism. The ammonium transceptors from *S. cerevisiae* (Mep2), *C. albicans* (Mep2), and *C. neoformans* (Amt2) regulate morphology changes in response to ammonium import.

Fungal pathogens prefer certain carbon sources for rapid uptake and metabolism to provide energy for growth and host colonization. The main carbon sources available in a host during fungal infection are glucose, lactate, and acetate (Ries et al., 2018). In addition, inositol can also be utilized as a carbon source by some fungi, including *Cryptococcus neoformans*. Multiple carbon sources and fungal sensory systems have been reported in pathogenic yeasts (Figure 1).

### Sugar Transporter Homologs Functioning as Sugar Sensors: Transceptors

Glucose is a preferred carbon and major energy source for most cells. The glucose sensing and signaling networks have been well-characterized in *Saccharomyces cerevisiae* (Kim and Johnston, 2006; Santangelo, 2006). In *S. cerevisiae*, two glucose transceptors, Snf3 and Rgt2, sense extracellular glucose levels and regulate the expression of the hexose transporter gene family, which comprises more than 20 genes. Snf3 is a high-affinity glucose sensor and is activated by low glucose levels, while Rgt2 is a low-affinity glucose sensor that is activated by high glucose levels. Both Snf3 and Rgt2 have long C-terminal cytoplasmic tails that interact with casein kinase I (Yck1 and Yck2) and with two transcription regulator proteins (Mth1 and Std1). Although Snf3 and Rgt2 do not transport glucose themselves (Ozcan et al., 1998), it is speculated that glucose binding leads to their conformational changes that activate casein kinase I. In the absence of glucose, Mth1 and Std1 serve as corepressors with the master transcription repressor Rgt1. These three proteins form a complex that binds to the promoter of hexose transporters to repress their expression (Moriya and Johnston, 2004). When glucose is available, Mth1 and Std1 are phosphorylated by casein kinase I and ubiquitinated by the SCF (Skp1-Cullin-F-box protein) E3 ubiquitin ligase Grr1, which leads to their degradation by the 26S proteasome. Depletion of the corepressors dissociates Rgt1 and relieves repression of hexose transporter gene transcription (reviewed in Santangelo, 2006). Hexose transporter genes are the primary regulatory targets of this glucose sensing mechanism in yeast, and this regulation optimizes the expression levels of high- and low-affinity hexose transporters.

The long cytoplasmic tail of Snf3 and Rgt2 plays an important role in glucose sensing by bringing Mth1 and Std1 to Yck1 in the membrane. These cytoplasmic tails can be fused to other sugar transporters and overexpression of these chimeric proteins can derepress expression of hexose transporter genes (Ozcan et al., 1998). Furthermore, overexpression of a membrane-targeted form of the cytoplasmic tail alone leads to constitutive activation of the glucose sensing pathway (Dlugai et al., 2001). However, the tails do not seem to be essential for glucose signaling, because overexpression of an Rgt2 allele lacking the tail also activates hexose transporter (Hxt1) expression (Moriya and Johnston, 2004). It is possible that Rgt2 may stimulate Yck1 through an interaction independent of Yck1 binding to the cytoplasmic tail of Rgt2.

Among over 20 hexose transporter homologs in *Candida albicans* (Fan et al., 2002), Hgt4 has been identified as a high-affinity glucose transceptor (Brown et al., 2006). Hgt4 shares sequence and structure similarity with other hexose transporters, with the exception of a long C-terminal tail containing 254 amino acids (aa), similar to Snf3 and Rgt2 in *S. cerevisiae*. Hgt4 is required for glucose induction of other hexose transporter genes, including *HGT12, HXT10*, and *HGT7*. Mutagenesis demonstrated that Hgt4 is required for fungal growth on fermentable sugars, such as fructose, mannose, and glucose. Hgt4 is also required for the yeast-to-hyphal morphological switch as well as fungal virulence, demonstrating that *C. albicans* cells need to sense and regulate sugar levels for filamentous growth during infection. The Hgt4-mediated regulatory mechanism of glucose repression in *C. albicans* is conserved with its counterpart in *S. cerevisiae*, and involves the casein kinase Yck2, corepressor Std1, and Rgt1 (Kim and Johnston, 2006; Sabina and Brown, 2009).

Despite the importance of glucose sensing and utilization in fungal development and virulence, glucose sensing is less well-understood in *C. neoformans*. There are about 50 transporters that share high sequence similarity with known hexose transporters (Liu et al., 2013b). Although some of them may transport sugars other than glucose, it remains elusive how this large family of hexose transporters is regulated. Among these hexose transporter homologs, Hxs1 and Hxs2 share the highest sequence identity with *S. cerevisiae* Rgt2 and Snf3 glucose transceptors, but neither of them have a long C-terminal tail (Liu et al., 2013b). The expression of Hxs1 is negatively regulated by glucose levels, and mutagenesis analysis showed that Hxs1 is required for efficient glucose uptake and fungal growth under low glucose conditions. Hxs1 is also required for fungal virulence in a murine model of systemic cryptococcosis. However, Hxs1 only modestly regulates the expression of other hexose transporters and it still has glucose uptake activity (Liu et al., 2013b). It is possible that Hxs1 has dual functions as both a glucose sensor and glucose transporter. On the other hand, the function of Hxs2 remains undefined. The downstream regulatory mechanism of glucose repression has not been characterized in detail in *C. neoformans*, but the function of the two casein kinase I, Cck1 and Cck2, has been studied. While the function of Cck2 is unknown, Cck1 is required for cell integrity and stress response by regulating the phosphorylation of Mpk1 and Hog1 mitogen-activated protein kinases (MAPKs) and also essential for fungal virulence (Wang et al., 2011b).

### GPCRs in Glucose Sensing

The role of GPCRs in glucose sensing has been well-characterized in *S. cerevisiae*. The GPCR receptor Gpr1 encodes a protein containing over 800 aa with a large third cytoplasmic loop and a long C-terminal tail. Gpr1 and its homologs in other fungi share limited sequence homology with the other defined GPCR classes (Attwood and Findlay, 1994; Kolakowski, 1994), and are grouped as a novel evolutionarily distinct GPCR class. Gpr1 senses glucose to activate the G-protein α subunit Gpa2 and regulates yeast cell size and pseudohyphal growth. The binding of glucose with Gpr1 leads to a conformational change that activates Gpa2, which in turn activates adenylyl cyclase to convert ATP into cAMP (Xue et al., 1998; Kraakman et al., 1999; Lorenz et al., 2000b; Rolland et al., 2002; Lemaire et al., 2004). cAMP then binds to the regulatory subunit (Bcy1) of protein kinase A (PKA) and thereby releases the catalytic subunits (Tpk1, 2, 3) of PKA to phosphorylate downstream target proteins. Interestingly, Gpr1 also senses sucrose to activate the Gpa2-cAMP pathway, and the affinity of Gpr1 for sucrose is much higher than for glucose. The half-maximal effective concentration (EC_50_) for sucrose is around 0.5 mM, compared to approximately 20 to 30 mM for glucose to activate the Gpa2-cAMP pathway. Meanwhile, mannose acts as an antagonist for glucose and sucrose in Gpr1 activation (Versele et al., 2001; Lemaire et al., 2004).

The Gpr1 protein sequence is conserved in *C. albicans*. Similar to *S. cerevisiae*, the *C. albicans* Gpr1 receptor binds to Gpa2 to activate G protein signaling, which in turn activates the cAMP-PKA signaling pathway (Miwa et al., 2004; Maidan et al., 2005a). Gpr1 is important for filamentous growth on solid media, but not in lipid medium (Miwa et al., 2004). However, the role of *C. albicans* Gpr1 in glucose sensing remains unclear. Some studies showed that Gpr1 and Gpa2 do not have a role in glucose-induced cAMP signaling and may not be involved in glucose sensing (Maidan et al., 2005a). Instead, deletion mutants of Cdc25 or Ras2 lack glucose-induced cAMP signaling, suggesting that the Cdc25-Ras2 branch is instead responsible for glucose sensing in *C. albicans*. It is possible that amino acids such as methionine are the ligand for Gpr1 in *C. albicans*, as methionine can trigger Gpr1 internalization and methionine induction of hypha formation on solid media requires a functional Gpr1 (Maidan et al., 2005a). However, the effect of methionine on hypha formation requires the presence of a low level of glucose in the medium. Therefore, it remains possible that Gpr1 may sense methionine, glucose, or both (Maidan et al., 2005b).

In *C. neoformans*, glucose sensing and utilization is critical for its development and virulence. In addition to being utilized as a preferred energy source for cell growth, glucose is required for capsule production both as a substrate and a signaling molecule. Glucose induces capsule enlargement through the Gpa1-cAMP-PKA signal transduction pathway, which plays a central role in fungal virulence (Alspaugh et al., 1997; Bahn et al., 2007). Although the cAMP-regulated PKA pathway is largely conserved, there is no Gpr1 homolog. Two GPCRs, Gpr4 and Gpr5, share structural similarity with Gpr1. Similar to Gpr1, Gpr4 encodes a large protein containing more than 800 aa with a long third cytoplasmic loop and C-terminal tail, but is not important for glucose sensing because glucose-mediated cAMP signaling activation is independent of Gpr4 function (Xue et al., 2006). Rather, *gpr4*Δ mutants exhibit defects related to methionine-induced morphogenesis, which is similar to what has been observed in *Candida* Gpr1. Gpr5 is a smaller protein that shares high sequence identity with Gpr4, and its mutant has shown defects in “Titan” cell production (Okagaki et al., 2011). The *gpr4*Δ *gpr5*Δ double mutants have even more pronounced defects in cell size regulation, suggesting these two GPCRs have overlapping functions. It is possible that Gpr5 may be involved in sensing carbon, including glucose. Gpr4 and Gpr5 have been shown to interact with Gpa1 to activate the cAMP-PKA pathway and control cell size and capsule (Okagaki et al., 2011).

### Sensing Other Carbon Sources

Inositol is a small carbohydrate molecule that functions as an essential structural and signaling molecule in eukaryotes, including fungi (Fisher et al., 2002; Xue, 2012). The myo-inositol transporter gene family belongs to the sugar transporter superfamily and may also play important roles in myo-inositol sensing in fungi. High sequence similarity within this gene family suggests that these genes likely evolved from a common ancestor. There are two myo-insoitol transporters (ITRs) in *S. cerevisiae* (Nikawa et al., 1991, 1993) and in *C. albicans* (Jin and Seyfang, 2003; Chen et al., 2008), but no inositol sensor has been identified in these yeast organisms.

Inositol seems to play a significant role in *C. neoformans* development and pathogenicity. It can be used as a sole carbon source (Healy et al., 1977) and can also stimulate *Cryptococcus* mating (Xue et al., 2007). As one of the most abundant metabolites in the mammalian brain (Fisher et al., 2002), inositol utilization is required for *C. neoformans* virulence in murine infection models by promoting brain infection (Xue et al., 2010; Wang et al., 2011a; Liu et al., 2013a, 2014). Therefore, *C. neoformans* likely utilizes the abundant inositol available inside the mammalian brain for its pathogenicity. Inositol can also stimulate *C. neoformans* capsule growth, which may contribute to its role in fungal virulence. The cryptococcal genome reflects the evolutionary adaptations associated with the expanded role of inositol in this organism. In particular, *C. neoformans* contains an unusually large number of ITRs that consists of more than 10 members (Xue et al., 2007, 2010). Functional analysis of the *ITR* gene family demonstrated that two members (Itr1a and Itr3c) have high inositol uptake activity, while the functions of the other members remain undefined. Of these, Itr1a could be a possible inositol transceptor because it does not show uptake activity in a yeast heterologous system, but it does regulate other *ITR* gene expression and the *itr1a*Δ single mutant exhibits defects in mating, hyphal production, and sporulation (Xue et al., 2010; Wang et al., 2011a). Therefore, an inositol transceptor may exist in *C. neoformans*. Importantly, fungal inositol transporters are proton-dependent symporters, which are pharmacokinetically different from the sodium-dependent human inositol transporters (Jin and Seyfang, 2003). Therefore, fungal inositol transporters may be developed as a valuable antifungal drug target.

Two- and three-carbon (C_2_ and C_3_) substrates are another important carbon sources for fungal pathogens. In *C. albicans*, non-fermentative carbon assimilation by glyoxylate and gluconeogenic pathways, which metabolizes C_2_ and C_3_ compounds such as acetate and lactate for glucose production, is critical for its early interaction with host immune cells, in which preferred carbon source such as glucose is limited (Lorenz and Fink, 2001; Barelle et al., 2006). For systemic infection, however, the fermentative glycolysis is the major carbon assimilation pathway for *C. albicans* (Barelle et al., 2006). In *C. neoformans*, the gluconeogenic pathway, but not the glyoxylate pathway, is required for the initial establishment of infection in the lungs (Rude et al., 2002; Panepinto et al., 2005; Price et al., 2011). For colonization and proliferation of *C. neoformans* in the central nervous system, however, glycolysis, but not gluconeogenesis, is critical (Price et al., 2011). Therefore, coordinated regulation of non-fermentative and fermentative carbon assimilation pathways depending on different infection stages appears to be essential for the pathogenicity of the two pathogenic yeasts.

In addition to sugars that are commonly utilized as carbon sources for energy and substrates, fungi also sense other carbon compounds as signaling molecules, mainly alcohol related carbons, to regulate cellular function. Alcohol-related carbon sensing has been reported mostly in *S. cerevisiae* and *C. albicans* and is less studied in *C. neoformans. S. cerevisiae* senses fusel alcohols, such as 1-butanol and isoamyl alcohol, to regulate differentiation of haploid cells. This involves binding of the transcription factor Ste12 to filamentation-specific genes in a Tec1-dependent mechanism (Dickinson, 1996; Lorenz et al., 2000a; Zeitlinger et al., 2003; Chen and Fink, 2006). Butanol also induces pseudohyphal morphology, even in liquid medium, which involves the Swe1-dependent morphogenesis checkpoint and differs from nitrogen-limitation-induced pseudohyphal growth (Martinez-Anaya et al., 2003). Ethanol stimulates hyperfilamentation in diploid cells in a MAPK-dependent manner (Lorenz et al., 2000a; Dickinson, 2008). In addition, aromatic alcohols (such as tryptophol and phenylethanol) secreted by yeast cells may function as quorum sensing molecules and stimulate filamentous growth in response to both cell density and nutritional conditions of the surrounding environment through a Flo11-dependent mechanism in *S. cerevisiae* (Chen and Fink, 2006). These autoregulatory molecules appear to function in a species-specific manner, because they only trigger the morphological switch in *S. cerevisiae*, but not in *C. albicans* (Chen and Fink, 2006).

Other alcohol-related quorum sensing molecules, such as farnesol and tyrosol, have been extensively studied in *Candida* species (Zhang and Dong, 2004; Hogan, 2006; Nickerson et al., 2006). Farnesol accumulation at the early stationary phase triggers inhibition of both yeast growth and filamentation by blocking GTPase activation, mitosis, and DNA replication in *C. albicans* (Uppuluri et al., 2007). Farnesol is also a virulence factor and it inhibits macrophage function during fungal-host interactions (Navarathna et al., 2007). In contrast, tyrosol stimulates fungal filamentation at all growth stages. Thus, the quorum-sensing process is under complex positive and negative regulation in response to environmental conditions (Chen et al., 2004). Fungal receptors that sense these quorum sensing molecules are largely unknown, although the Chk1 histidine kinase has been reported to play a role in sensing farnesol in *C. albicans* (Kruppa et al., 2004).

The C_3_ compound lactate was also shown to be a signaling molecule in *C. albicans*. Exposure to L-lactate, but not D-lactate, triggers β-glucan masking in *C. albicans* by regulating cell wall genes, which allows the pathogen to evade host immune detection (Ballou et al., 2016). Notably, this process is regulated by the Gpr1 GPCR and the Crz1 transcription factor, but is independent of lactate metabolism. Although this lactate-induced β-glucan masking process is conserved in other *Candida* clade (Ballou et al., 2016), it remains unknown in *C. neoformans*.

## Nitrogen Sensing in Fungal Pathogenicity

Fungal pathogens sense nitrogen levels to control their rate of growth and changes in their morphology, processes that are important for host infection. The extracellular sensing of nitrogen containing compounds occurs via transceptors and GPCRs. Although transceptors are potential antifungal targets due to their cellular localization and their control of major signaling pathways, we currently lack an understanding of the molecular mechanisms involved in transceptor mediated signaling. To explore their therapeutic and economic potential, the molecular mechanisms that underpin fungal transceptor signaling need to be fully characterized.

### Transceptors That Control the PKA Pathway

The PKA pathway regulates processes that are associated with cell growth in *S. cerevisiae*. Under nutrient replete conditions that include a fermentable carbon source, *S. cerevisiae* grows quickly and exhibits high PKA activity. However, if an essential nutrient is missing from the medium, cells are arrested, enter the stationary phase, and display phenotypes associated with low PKA activity, including high expression of stress-related genes and the production of stored carbohydrates. Under these conditions nutrient replacement results in rapid induction of PKA activity. The signaling pathway that regulates this response is known as the fermentable-growth-medium induced pathway (FGM pathway) (Thevelein, 1994). Activation of the FGM pathway is dependent on a family of transceptors that import amino acids (Gap1), ammonium (Mep1 and Mep2), phosphate (Pho84), sulfate (Sul1 and Sul2), iron (Ftr1), and zinc (Zrt1) (Figure 1) (Donaton et al., 2003; Giots et al., 2003; Van Nuland et al., 2006; Kankipati et al., 2015; Schothorst et al., 2017).

A well-studied model of a PKA regulating transceptor is the general amino acid permease (Gap1) of *S. cerevisiae*, which is a low affinity permease that imports a broad range of amino acids in cells grown under nitrogen-limiting conditions (Jauniaux and Grenson, 1990; Donaton et al., 2003). Two sites that are important for amino acid binding (Ser388 and Val389) have also been identified in Gap1 (Van Zeebroeck et al., 2009). When ammonium is added to yeast cells growing with proline as the only nitrogen source, Gap1 is rapidly internalized and degraded via a pathway involving the Npi1/Rsp5 ubiquitin ligase (Springael and Andre, 1998). The Ras2/cAMP/PKA pathway may be involved in the ubiquitin-dependent degradation of Gap1 (Garrett, 2008). Gap1 biogenesis is coupled to sphingolipid biosynthesis, which produces a sphingolipid microenvironment essential for the normal conformation, function, and ubiquitination of Gap1 (Lauwers et al., 2007). The activation of the FGM pathway by different nutrient transceptors suggests that they utilize a common signaling mechanism and a number of experimental findings are consistent with nutrient sensing involving an aspect of transporter function rather than changes in internal nutrient metabolism. For example, the FGM pathway is activated in mutants that are unable to metabolize the signaling nutrient or by transceptor-mediated uptake of a non-metabolizable nutrient analog (Donaton et al., 2003; Giots et al., 2003; Van Nuland et al., 2006). Importantly, amino acid substitutions have been identified that separate the transport and signaling functions of transceptors. Mutations of the predicted proton-binding sites within the Pho84 and the Sul1/2 nutrient/proton symporters result in a loss of nutrient transport but do not impact signaling, presumably due to continued substrate binding to the transceptor (Samyn et al., 2012; Kankipati et al., 2015).

A favored hypothesis is that the PKA-regulating transceptors act in a way that is analogous to GPCRs (Holsbeeks et al., 2004; Thevelein and Voordeckers, 2009). This model predicts that conformational changes within the transceptor following substrate binding and/or transport alter the interaction between the transceptor and a signaling partner that regulates the FGM pathway. This potential mechanism is consistent with the hypothesis that transceptors represent intermediates in the evolution of receptors from nutrient transporters and is supported by the existence of the yeast glucose sensors Snf3 and Rgt2 that have homology with nutrient transporters but no transport function (Ozcan et al., 1996; Didion et al., 1998; Thevelein and Voordeckers, 2009). In *S. cerevisiae*, another non-transporting transceptor plays an important role in amino acid sensing (Didion et al., 1998; Klasson et al., 1999; Forsberg and Ljungdahl, 2001; Wu et al., 2006). In this system, Ssy1 is a transceptor that senses amino acids without transporter activity. Ptr3 and Ssy5 function downstream of Ssy1 and physically interact with Ssy1 to form a signaling complex (Forsberg and Ljungdahl, 2001). Binding of amino acids to Ssy1 activates the Ssy5 protease which, in turn, proteolytically activates the latent transcription factors Stp1 and Stp2 to induce expression of genes encoding amino acid metabolizing enzymes and amino acid permeases (Andreasson et al., 2006). Ssy1 senses extracellular amino acids and activates Ptr3 hyperphosphorylation, which is dependent on SCF (Skp1-Gullin-F-box)-Grr1 E3 ligase complex function, but independent of Ssy5 function. Deletion mutations of Grr1, the F-box protein of this E3 ligase complex, block amino acid-induced Ptr3 hyperphosphorylation (Liu et al., 2008).

**FIGURE 2:**
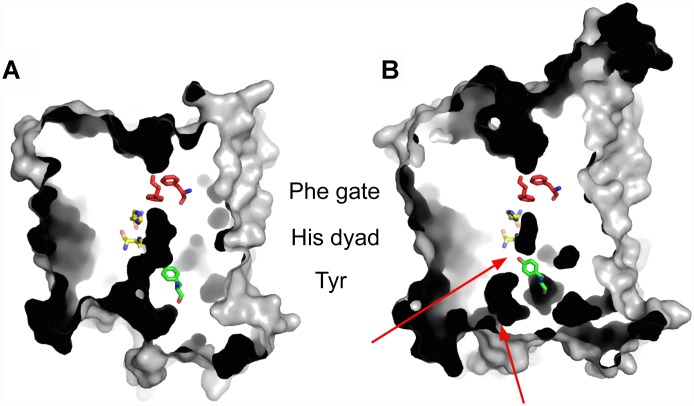
The crystal structure of ammonium transceptor Mep2. Slab views from the membrane plane, showing *E. coli* AmtB **(A)** and *S. cerevisiae* Mep2 **(B)**. The two-tier channel block in *Sc* Mep2 is indicated by red arrows. In *Sc* Mep2 Tyr53 makes a strong hydrogen bond with His348, which is one of two histidine residues within the ammonium-conducting channel. The dark areas represent internal cavities and channels.

Evidence that conformational changes are associated with transceptor function comes from recent X-ray crystal structures of the Mep2 ammonium transceptors from *S. cerevisiae* and *C. albicans* (Figure 2) (van den Berg et al., 2016). Mep2 proteins are evolutionarily conserved members of the Amt/Mep/Rh family of ammonium transporters. Several structures of this class of proteins have been determined from bacteria as well as a structure of a human Rhesus protein (Khademi et al., 2004; Zheng et al., 2004; Andrade et al., 2005; Javelle et al., 2006; Gruswitz et al., 2010). They all form stable trimers with each monomer having an extracellular ammonium-binding site, a pair of conserved phenylalanine residues that gate a central narrow hydrophobic pore through which ammonium is conducted. However, there are structural differences that distinguish the fungal Mep2 transceptor from other ammonium transporters, and which may relate to the potential transceptor signaling mechanism (van den Berg et al., 2016). Fungal Mep2 transceptors have a closed conformation with a two-tier block of the central pore whereas all other ammonium transporters with known structures have an open pore. It is likely that the closed state of Mep2 prevents ammonium import, suggesting that the Mep2 transceptor must undergo significant conformational changes if ammonium conductance is to occur. Mep2 is activated via the phosphorylation of a serine residue within the cytoplasmic C-terminal domain (CTD) by the target of rapamycin complex 1 (TORC1) regulated Npr1 kinase, and this is hypothesized to open the ammonium-conducting pore (Boeckstaens et al., 2014; van den Berg et al., 2016). Conversely, the dephosphorylation of this site by the Psr phosphatases blocks ammonium transport (Boeckstaens et al., 2014). The structure of a Mep2 variant with a phosphomimetic mutation of this regulatory serine residue shows a large conformational change within the CTD that includes the formation of a novel 12-residue helix (van den Berg et al., 2016). This structural change is predicted to cause a shift of the CTD toward the main body of the transceptor to open the ammonium channel. It is conceivable that this conformational change also regulates binding of a signaling partner to the Mep2 CTD. While there is evidence that transceptors undergo conformational changes that are linked to signaling, the identification of transceptor signaling partners has not been successful. A split-ubiquitin two-hybrid screen identified proteins that interact with Mep2 and Gap1 and that are involved in different cellular processes (Van Zeebroeck et al., 2011). However, no signaling protein that links Mep2 or Gap1 directly to the FGM pathway was identified.

Currently less is known about the role of transceptors in *C. albicans* and *C. neoformans.* Ssy1, Ptr3, and Ssy5 homologs in *C. albicans* have been reported to function in a pathway that senses amino acids and is important for virulence in the host (Brega et al., 2004; Martinez and Ljungdahl, 2005). There are also six Gap1 homologs in *C. albicans*, and three (Gap1, Gap2, and Gap6) have transceptor activity to sense amino acids because they can rapidly activate the PKA pathway (Kraidlova et al., 2011). In *C. neoformans* eight amino acid permease homologs have been identified based on genome information, but their potential role in amino acid sensing has not been analyzed (Fernandes et al., 2015). Also the expression of *CFT1* and *CFT2*, two *C. neoformans* homologs of iron transceptor Ftr1, are also regulated by PKA activity, and both proteins are required for fungal virulence (Jung et al., 2008). Yet, it is not clear whether either one functions as an iron sensor.

### Ammonium Transceptors That Regulate Fungal Morphology

An ammonium transceptor dependent signaling system in dimorphic yeast regulates a switch in morphology in response to limiting nitrogen levels (Figure 1) (Lorenz and Heitman, 1998; Smith et al., 2003; Biswas and Morschhauser, 2005; Rutherford et al., 2008b). In *S. cerevisiae*, this regulates a switch to a filamentous form of growth known as pseudohyphal growth (Gimeno et al., 1992). Pseudohyphal growth is a Mep2-dependent process and results in *S. cerevisiae* forming chains of elongated cells that allow the yeast to forage for nutrients when they utilize a poor or limiting nitrogen source (Lorenz and Heitman, 1998; Boeckstaens et al., 2007). A number of signal transduction pathways regulate pseudohyphal growth and include the PKA, MAPK, sucrose non-fermentable, and TORC1 signaling pathways (Cullen and Sprague, 2012). Constitutively active components of the PKA or MAPK pathways restore pseudohyphal growth in a mutant lacking Mep2, suggesting that this transceptor may regulate these pathways during the dimorphic switch (Lorenz and Heitman, 1998; Biswas and Morschhauser, 2005; Rutherford et al., 2008a). The transport and pseudohyphal signaling functions of Mep2 may be linked as a hyperactive transporting and signaling Mep2 variant has been identified and Mep2 variants that do not transport ammonium but are correctly expressed and localized do not induce pseudohyphal growth (Marini et al., 2006; Boeckstaens et al., 2007; Rutherford et al., 2008a). Mep2 separation-of-function variants that transport ammonium but do not induce pseudohyphal growth also establish that ammonium sensing is not a consequence of changes in internal nitrogen metabolism (Van Nuland et al., 2006; Boeckstaens et al., 2008; Rutherford et al., 2008a). Therefore, ammonium sensing and the consequent regulation of pseudohyphal growth are dependent on an aspect of ammonium conductance through Mep2. Mep2 transceptor homologs also regulate morphological changes in *C. albicans* and *C. neoformans* (Smith et al., 2003; Biswas and Morschhauser, 2005; Rutherford et al., 2008b). Similar to the transceptor-mediated regulation of the FGM pathway, two models of Mep2 signaling during pseudohyphal growth have been proposed. In the first, Mep2-dependent ammonium transport is predicted to cause changes in cytosolic pH that is then sensed by an internal pH-responsive mechanism (Boeckstaens et al., 2008). This model is based on differences in the optimal pH for ammonium transport between Mep2 and two paralogous non-signaling ammonium transporters, Mep1 and Mep3 (Boeckstaens et al., 2008). Furthermore, a link between pH and polarized growth has been identified in other fungal systems (Bowman et al., 1997; Martinez-Espinoza et al., 2004; Vylkova et al., 2011). The second model of Mep2 signaling involves this transceptor acting analogous to GPCRs by physically interacting with and regulating a signaling partner as has been proposed for regulation of the FGM pathway (Lorenz and Heitman, 1998).

Ammonium metabolism can promote fungal infection as its export can alkalinize the external environment. *C. albicans* secrets ammonium to raise external pH and induce its filamentous form (Vylkova and Lorenz, 2014). Similarly *C. neoformans* uses urea as a nitrogen source resulting in the production and secretion of ammonium, which can have diverse impacts on the host. Urease positive *C. neoformans* promote the induction of a non-protective Type 2 host-immune response (Osterholzer et al., 2009). Furthermore, urease is required for the ability of *C. neoformans* to cross the host blood brain barrier possibly by disrupting the integrity of the junctions between microvascular endothelial cells in the brain (Shi et al., 2010; Singh et al., 2013). Urease also modulates the pH of macrophage phagolysosomes (Fu et al., 2018). Consistent with these studies urease is required for the virulence of *C. neoformans* in a mouse model of infection (Cox et al., 2000). In the human host, urea is distributed evenly throughout the body at concentrations in the lower mM range (Liu et al., 2012; Singh et al., 2013). There is therefore a sufficient pool of this metabolite within humans to act as a nutrient source for a fungal pathogen and as a modulator of host pH.

### GPCRs and Amino Acid Sensing in Fungi

Amino acid sensing via GPCRs has not been reported in *S. cerevisiae*. In *C. albicans*, Gpr1 senses methionine to regulate the yeast-to-hypha transition on solid medium in the presence of carbon sources such as glucose (Maidan et al., 2005a). It is currently unclear whether Gpr1 directly senses extracellular methionine, intracellular amino acids, or both. It is possible that Gpr1 may sense both methionine and glucose.

Similar to Gpr1 in *C. albicans*, Gpr4 in *C. neoformans* has also been found to sense amino acids and activate signaling by the Gα protein Gpa1 signaling, which in turn activates cAMP-PKA signaling (Xue et al., 2006). Methionine induces the internalization of a Gpr4-DsRED fusion protein and also induces transient cAMP production in *C. neoformans*, and both are blocked by *gpr4Δ* mutations. A low concentration of methionine in the medium stimulates mating hyphae elongation in a Gpr4-dependent manner, but the role of methionine at a molecular level remains to be elucidated. Because Gpr4 contributes to but is not essential for the production of virulence factors controlled by cAMP signaling, and is not important for melanin production or virulence, additional upstream receptors other than Gpr4 may contribute to regulate Gpa1 functions. Activation of cAMP signaling by glucose and amino acids represents a nutrient coincidence detection system conserved in other pathogenic fungi.

### The TOR Pathway Is a Globally Conserved Nutrient Sensor

TOR (target of rapamycin) is a serine/threonine kinase of the phosphatidylinositol kinase-related kinase family, which shares conserved motifs (such as HEAT repeats, FAT, and FATC domains), and is structurally and functionally conserved in eukaryotes (Abraham, 2004). The TOR pathway is activated by a variety of environmental signals and acts as a central regulator of cell growth through the phosphorylation of substrates that stimulate anabolic processes and inhibit catabolic process such as autophagy (Wolfson and Sabatini, 2017). The discovery of the TOR signaling pathways began with studies that sought to identify the molecular targets for the novel immunosuppressive, antifungal natural product rapamycin, which was originally isolated from a strain of *Streptomyces hygroscopicus*, from the beaches of Easter Island (Sehgal et al., 1975). This drug had been discovered in screens for natural products at Wyeth-Ayerst to identify candidate antifungal agents with activity against *C. albicans* (Sehgal, 2003). Despite having very potent antifungal activity, studies on rapamycin were shelved when it was discovered that the compound caused bone marrow suppression (Martel et al., 1977). When FK506 was discovered as a novel immunosuppressant in a screen at Fujisawa Pharmaceutics for natural products that would inhibit a mixed lymphocyte response assay, rapamycin was appreciated to be structurally related to FK506 and studies began anew to study its immunosuppressive properties and potential (Kino et al., 1987).

The FK506 binding protein FKBP12 was purified from yeast and its mutants were found to be resistant to rapamycin and FK506 (Heitman et al., 1991a,b). Isolation of rapamycin resistant yeast mutants revealed mutations in three genes, FKBP12 and two novel genes (Heitman et al., 1991a), which were named TOR1 and TOR2 for Target of Rapamycin (Kunz et al., 1993; Helliwell et al., 1994), which were later found to form multiprotein complexes known as TORC1 and TORC2 (Loewith et al., 2002; Wedaman et al., 2003). The TORC1 complex is sensitive to rapamycin and involved in a wide range of functions, while the TORC2 complex regulates polarization of the actin cytoskeleton. Several years later studies from multiple groups converged to identify the mammalian ortholog of the yeast TOR proteins (Brown et al., 1994; Chiu et al., 1994; Sabatini et al., 1994; Sabers et al., 1995), now known as mTOR.

Rapamycin inhibits yeast cell growth by inhibiting the ability of yeast cells to appropriately sense and respond to nutrients, particularly nitrogen sources including ammonia and glutamine, for example. Three studies converged to reveal that the TOR pathway functions in nutrient sensing in yeast (Beck and Hall, 1999; Cardenas et al., 1999; Hardwick et al., 1999). These studies revealed two sets of genes to be profoundly disrupted in cells exposed to rapamycin. First, the genes encoding ribosomal proteins, ribosomal RNAs, and many other proteins and enzymes involved in translation were all found to be repressed by rapamycin. Concomitantly, a suite of genes that was induced in cells exposed to rapamycin including many genes encoding transporters for a variety of nitrogen sources, and enzymes and proteins involved in the utilization of alternative nitrogen sources, the so called nitrogen catabolite repression (NCR) response. These later genes are known to be regulated by Ure2 and Gln3, both of which participate in the TOR pathway governing the control of nutrient responsive gene suites. Thus, these studies were paradigmatic in showing that the TOR pathway orchestrates the growth of cells in response to nutrients. Several years later the mTOR pathway was found to similarly control nutrient responsive genes in mammalian cells (Peng et al., 2002). Much more is now known about the intricate pathways that enable TOR and mTOR to sense the availability of several different amino acids and nutrients, and to couple this to appropriate physiological outcomes (reviewed in Jewell et al., 2013; Goberdhan et al., 2016; Gonzalez and Hall, 2017; Wolfson and Sabatini, 2017).

The Tor proteins sense nutrient signals, including amino acids, and regulate a broad range of cell developmental and signaling processes, including ribosome biosynthesis, protein translation, starvation-related transcriptional regulation, and autophagy (Raught et al., 2001; Rohde et al., 2001; Rohde and Cardenas, 2003). Amino acids are sensed by the TORC1 pathway via a variety of molecular mechanisms including the GTP/GDP loading status for the Rag GTPases that are called the Ragulator Rag GTPase in mammals and the EGO (Ego1-Ego2-Ego3) complex in *S. cerevisiae*. In *S. cerevisiae*, amino acids activate the SEACAT (Seh1-associated complex subcomplex activating TORC1), which consists of Sec13, Seh1, Sea2, Sea3, and Sea4. The SEACAT binds and negatively regulates SEACIT (Seh1-associated subcomplex inhibiting TORC1), which functions as GTPase-activating proteins (GAPs) for Gtr1, a RAG family small GTPase that binds with EGO complex to tether the TORC1 complex to the vacuole membrane (Binda et al., 2009). The other GTPase Gtr2 is activated by the GAP protein Lst4 in response to amino acids. Amino acid sufficiency promotes the active conformation of the RAG GTPase heterodimer in which Gtr1 is loaded with GTP, while Gtr2 is loaded with GDP (Gong et al., 2011). The active Gtr1^GTP^-Gtr2^GDP^ heterodimer binds to Kog1 to activate TORC1 (reviewed by Gonzalez and Hall, 2017).

Overall, the core TOR complex is conserved among eukaryotes, while sensors that regulate the TOR pathway are likely more diverse. In mammalian cells, multiple amino acid sensors have been identified, including SLC38A9 as a putative arginine transceptor that positively regulates the mTOR pathway, the Sestrins (Sestrin 1 and 2) as a leucine sensor that functions as a negative regulator of the pathway, and CASTOR1 as a cytosolic arginine sensor for the pathway in a mechanism analogous to that of the Sestrins (reviewed by Saxton et al., 2016). However, similar amino acid sensors have not been identified in *S. cerevisiae* and most other fungi. It is possible that there are similar sensors in fungi, but with more sequence diversity. In yeast, leucine activates TORC1 via Gtr1. There is no Sestrin homolog and instead the leucyl-tRNA synthase has been shown to act as a cytosolic leucine sensor (Bonfils et al., 2012).

Rapamycin treatment triggers ubiquitination and degradation of some high-affinity amino acid transporters, such as the tryptophan permease Tat2 and the histidine permease Hip1, suggesting that Tor signaling promotes stability of these high-affinity, specialized transporter systems (Schmidt et al., 1998; Beck et al., 1999). On the other hand, Tor signaling also negatively regulates the stability of general amino acid permease systems like Gap1, and thus inversely regulates these two classes of amino acid permeases to balance the nutritional requirements of the cell (Beck et al., 1999). The serine/threonine kinase Npr1 has been recognized to mediate the regulation of these permeases by Tor (Schmidt et al., 1998; Raught et al., 2001).

Glutamine is a preferred nitrogen source and a key intermediate in yeast nitrogen metabolism, and its function is likely regulated by the TOR pathway. Glutamine depletion in yeast triggers nuclear localization and activation of the TOR-inhibited transcription factors Gln3, Rtg1, and Rtg3 (Crespo et al., 2002). Glutamine activation of TORC1 in yeast is independent of Gtr1, and rather requires the vacuolar membrane-associated phosphatidylinositol 3-phosphate binding protein Pib2 (Stracka et al., 2014; Kim and Cunningham, 2015). This nitrogen regulation is also mediated by the PP2A-like phosphatase Sit4. Many of the non-transcriptional effects of Tor, such as the initiation of translation and control of the stability of amino acid permeases, are also regulated via Sit4 (Di Como and Arndt, 1996; Cardenas et al., 1999; Rohde et al., 2004; Jacinto, 2007). Tor-mediated nutrient signaling also triggers nuclear translocation of Gln3 and a role of Golgi-to-endosome vesicular trafficking in TORC1-controlled nuclear translocation has been described (Puria et al., 2008).

In *S. cerevisiae*, Tor signaling is part of the complex signaling network that controls the yeast-to-filament switch in response to nitrogen limitation (Gimeno et al., 1992; Pan and Heitman, 1999; Cutler et al., 2001). Tor signaling involves the Sit4 protein phosphatase and is independent and parallel to the well-defined MAPK and cAMP-PKA pathways, but it is possible that signaling in response to nutrients involves crosstalk between these pathways (Schmelzle et al., 2004; Zurita-Martinez and Cardenas, 2005; Chen and Powers, 2006). Future studies are needed to understand the precise molecular mechanisms by which Tor regulates nutrient sensing and activation in fungi.

In addition to *S. cerevisiae*, TOR regulation of nutrient sensing has also been reported in *C. albicans*. Tor1 has been implicated in the negative regulation of filamentous growth in *C. albicans*. Inhibition of TORC1 results in the activation of the GATA transcription factor Brg1, which is involved in the regulation of hypha-specific genes and blocking the recruitment of the Nrg1-Tip1 transcriptional repressor complex (Lu et al., 2012; Su et al., 2013). Tor1 also plays a role in the regulation of adhesion gene expression in *C. albicans* (Bastidas et al., 2009). The Tor pathway in *C. albicans* has recently also been reported to regulate phosphate sensing that is dependent on the phosphate transporter Pho84 and the function of the TOR-activating small GTPase homologs Gtr1 and Rhb1 in nutrient responses has been characterized (Flanagan et al., 2017; Liu et al., 2017). The mechanisms of nutrient sensing by the TOR pathway in *C. neoformans* are less studied and remain to be understood.

## Stress Sensing in Fungal Pathogenicity

Fungal pathogens are confronted with a series of host-derived stresses during the whole infection process, including temperature shift, fluctuation of oxygen/carbon dioxide levels, osmotic stress, and oxidative/nitrosative stress. To cope with such a variety of environmental stresses, most fungi employs an evolutionarily conserved stress-activated MAPK pathway, commonly known as the high-osmolarity glycerol response (HOG) pathway. Although the HOG pathway was initially discovered as osmosensing system in *S. cerevisiae*, it was subsequently proved to be a general stress-sensing pathway in most fungi.

### Multicomponent Phosphorelay Systems

**FIGURE 3:**
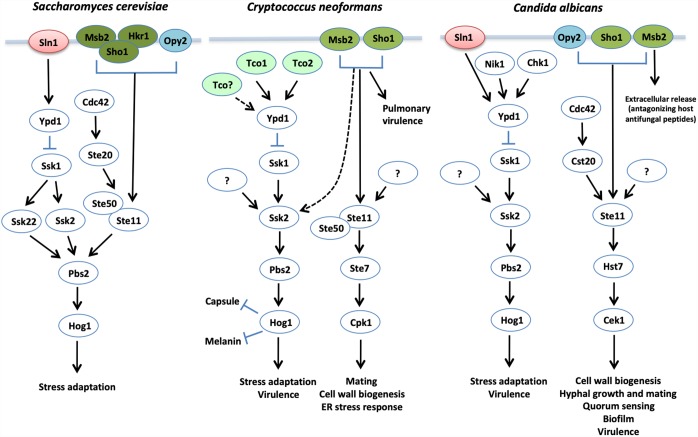
The proposed yeast HOG pathways. In *S. cerevisiae*, the Ssk2/22-Pbs2-Hog1 MAPK module is modulated by two main upstream branches: the Sln1-Ypd1-Ssk1 phosphorelay system and Msb2/Hkr1/Sho1/Opy2 signaling branches. In contrast, the Ssk2-Pbs2-Hog1 MAPK module is mainly regulated by the multicomponent phosphorelay system in both *C. neoformans* and *C. albicans*. In *C. neoformans*, the Msb2/Sho1 branch pathway plays a minor role in phosphorylating Hog1 in the absence of Ssk1, and functions in phosphorylating Cpk1 for cell wall biogenesis. Although Msb2 and Sho1 are involved in mating process of *C. neoformans*, they are not directly involved in Cpk1 phosphorylation. In *C. albicans*, there is no evidence that the Sho1/Msb2/Opy2 branch pathway regulates the Hog1 MAPK module directly.

Fungi utilize phosphorelay systems to sense and respond to a variety of environmental stresses. These phosphorelay systems are widely conserved in prokaryotes and generally consists of two signaling components, sensor histidine kinase (HK), composed of His kinase A and histidine kinase-like ATPase domains serving as an autophosphorylation catalytic core, and its downstream response regulator (RR) (Zschiedrich et al., 2016; Tiwari et al., 2017). In contrast, most fungi contain multicomponent phosphorelay systems, which consist of sensor hybrid histidine kinases (HHKs), histidine-containing phosphotransfer (HPt) protein, and RRs (Figure 3) (Santos and Shiozaki, 2001; Matsushita and Janda, 2002; Laub and Goulian, 2007; Bahn, 2008). The HHKs consist of an HK domain and an aspartate-containing receiver domain (RD). Upon external cues, the HHK undergoes autophosphorylation at the histidine residue in the HK domain and relays its phosphate group to the aspartate residue in the RD. Subsequently, the phosphate group is relayed to the histidine residue in the HPt protein, which is then transferred to the aspartate residue in RRs (Santos and Shiozaki, 2001; Matsushita and Janda, 2002; Laub and Goulian, 2007; Bahn, 2008). Most fungi contain two types of RRs: one RR, similar to a bacterial RR, has a DNA-binding domain and transcriptionally activates effector genes and the other RR activates another signaling cascade, including a Hog1 MAPK module. *S. cerevisiae* also contains two types of RRs, Ssk1 and Skn7, and the former localizes to the cytoplasm whereas the latter is enriched in the nucleus (Lu et al., 2003). The HPt protein, Ypd1, shuttles between the cytoplasm and nucleus to relay its phosphate group to Ssk1 and Skn7, respectively, depending on the type of eliciting stresses (Lu et al., 2003).

The most notable feature of the fungal phosphorelay system is a tremendous diversity of HHKs in both number and domain structures (Bahn, 2008; Herivaux et al., 2016). *S. cerevisiae* contains only a single HHK (Sln1), while some filamentous fungi contains more than 10 HHKs. Although all HHKs commonly contain at least a single HK and one RD, their N-terminal regions contain highly diverse protein domains, which are proposed to play distinct roles for each HHK in sensing different signals and regulating the activity of the HK and RD domains.

*Cryptococcus neoformans* and *Candida albicans* contain seven and three HHKs, respectively (Bahn, 2008). This varying number of HHKs may reflect differential host biological niches at which these two fungal pathogenic yeasts reside during infection. *C. neoformans* HHKs were named Tco1 to Tco7 (Two-component proteins 1–7) (Bahn et al., 2006). Tco1 contains seven HAMP (Histidine kinases-Adenylyl cyclases-Methyl accepting proteins-Phosphatases) domains with an extended N-terminal region (Bahn et al., 2006). Notably, *C. neoformans* contains two unique dual HHKs, Tco2 and Tco4, which harbor two HK-RD domains in a single polypeptide (Bahn et al., 2006; Bahn, 2008). Since their first discovery in *C. neoformans*, a recent bioinformatics analysis showed that the dual HHKs are observed only in certain basidiomycetous fungi (Lavin et al., 2014). Tco3 contains GAF (cGMP-specific phosphodiesterases-Adenylyl cyclases-FhlA) and PHY (phytochrome) domains. Tco5 is the only TM domain-containing HHK. Both Tco6 and Tco7 are GAF-containing HHK. In stark contrast to Sln1, which is essential for viability of *S. cerevisiae*, none of the Tco HHKs are essential for *C. neoformans* (Bahn et al., 2006; Lee et al., 2016). Among these, Tco1 and Tco2 are two major HHKs in *C. neoformans* (Bahn et al., 2006). Both HHKs are involved in recognizing fludioxonil, a phenylpyrrole class of fungicide (Bahn et al., 2006). However, Tco1 and Tco2 also have distinct roles. Tco1 is involved in hypoxia sensing, melanin production, the mating process, and virulence, whereas Tco2 is involved in response and adaptation to osmotic and oxidative stress and toxic metabolites (Bahn et al., 2006; Chun et al., 2007).

*Candida albicans* has three HHKs: Sln1, Nik1/Cos1, and Chk1 (Alex et al., 1998; Nagahashi et al., 1998; Yamada-Okabe et al., 1999). Although *C. albicans* Sln1 can functionally replace *S. cerevisiae* Sln1 and is indeed involved in osmosensing, Sln1 is not essential for viability of *C. albicans* (Nagahashi et al., 1998). The most notable function of these HHKs is the regulation of the morphological transition, which is a crucial virulence factor for *C. albicans* (Alex et al., 1998; Nagahashi et al., 1998; Yamada-Okabe et al., 1999). Deletion of *SLN1, NIK1*, or *CHK1* causes defects in hyphal development and thereby attenuates the virulence of *C. albicans* (Yamada-Okabe et al., 1999). However, *CHK1* deletion partially restores filamentation and virulence in the *sln1*Δ and *nik1*Δ mutants, suggesting that complex cross-talk may occur among these HHKs (Yamada-Okabe et al., 1999).

Regardless of the remarkable diversity of HHKs, most fungi contain only one or two HPt proteins. Therefore, it is still puzzling how different environmental signals sensed by varying HHKs are distinguished by such a small number of HPt proteins. In *C. albicans* and *C. neoformans*, a single HPt (Ypd1) has been discovered. In *S. cerevisiae*, Ypd1 is essential because its absence leads to constitutive dephosphorylation of Ssk1, which overactivates the Ssk2/22-Pbs2-Hog1 MAPK module (Posas et al., 1996). Ypd1 is similarly essential in *C. neoformans*, but *YPD1* deletion is feasible in the *hog1*Δ mutant background, suggesting that *YPD1* deletion also hyperactivates Hog1 in the pathogen (Lee et al., 2011). *C. albicans YPD1* can functionally replace *S. cerevisiae YPD1*. Similar to *S. cerevisiae* Ypd1, *C. albicans* Ypd1 also localizes to both the cytoplasm and the nucleus (Mavrianos et al., 2014). Notably, however, Ypd1 is not essential in *C. albicans*, although its deletion causes growth defects (Mavrianos et al., 2014). *YPD1* deletion causes constitutive Hog1 phosphorylation even under unstressed conditions, as expected, and flocculation through enhanced filamentation (Mavrianos et al., 2014).

*Cryptococcus neoformans* contains two most conserved classes of RRs, Skn7, and Ssk1 (Bahn et al., 2006). Ssk1 plays a major role in relaying the signal from Ypd1 to the Hog1 MAPK module as shown by the fact that the *ssk1*Δ mutant is phenotypically similar to the *hog1*Δ mutant (Bahn et al., 2006). However, Hog1 may have another upstream regulator as it can be phosphorylated in the absence of Ssk1 (Bahn et al., 2006). In contrast, Skn7, which contains a heat shock factor-type DNA binding domain at its N-terminus, plays both redundant and distinct roles with Hog1, but the Skn7-mediated signaling is largely independent of the HOG pathway (Bahn et al., 2006). Supporting this, *SKN7* deletion does not affect Hog1 phosphorylation and its related phenotypes, such as capsule production and mating efficiency (Bahn et al., 2006). In contrast, *C. albicans* contains three RRs: Ssk1, Skn7, and Srr1, which are localized to the cytoplasm, the nucleus, and the mitochondria, respectively (Calera et al., 2000; Singh et al., 2004; Mavrianos et al., 2013). Ssk1 is involved in oxidative stress and heat shock response, cell wall biogenesis, adherence, and filamentous growth, whereas Skn7 is involved in the morphological transition and oxidative stress response. Similar to the case in *C. neoformans*, Skn7 function is largely Hog1-independent in *C. albicans* (Singh et al., 2004). Srr1 is involved in hyphal development, stress resistance and virulence (Desai et al., 2011).

In *S. cerevisiae*, the dephosphorylated Ssk1 activates the autophosphorylation activity of the Ssk2/22 MAPK kinase kinase (MAPKKK) by interacting with the autoinhibitory domain of Ssk1 (Posas and Saito, 1998). Activated Ssk2/22 subsequently phosphorylates the MAPK kinase (MAPKK) Pbs2, which then phosphorylates the Hog1 MAPK. In contrast to *S. cerevisiae* that contains two MAPKKKs, Ssk2 and Ssk22, both *C. neoformans* and *C. albicans* contain a single Ssk2 ortholog. Pbs2 and Hog1 are also conserved in the two pathogens (Bahn et al., 2005). Regardless of the conserved Ssk2-Pbs2-Hog1 module, its regulatory mechanism appears to be divergent. Hog1 is not normally phosphorylated under unstressed conditions in both *S. cerevisiae* and *C. albicans*, but becomes rapidly phosphorylated in response to environmental stresses (Brewster et al., 1993; Smith et al., 2004). In *C. neoformans*, however, Hog1 is constitutively phosphorylated under unstressed conditions, but undergoes dephosphorylation in response to environmental stresses, such as osmotic shock (Bahn et al., 2005).

Transcriptome analysis revealed a plethora of Hog1 downstream effector genes in *C. albicans* and *C. neoformans* (Enjalbert et al., 2006; Ko et al., 2009). Among these, some of pathogenicity-related effectors are particularly notable. First, the Na^+^/ATPase efflux pump Ena1 was shown to be strongly induced by salt or osmotic stress in a Hog1-depenent manner and required for pH homeostasis in *C. neoformans* (Jung et al., 2012; Meyers et al., 2017). Deletion of *ENA1* completely abolishes the virulence of *C. neoformans* (Jung et al., 2012; Meyers et al., 2017). Second, a number of the genes involved in oxidative stress response, including a sulfiredoxin gene (*SRX1*), are strongly induced by peroxides in a Hog1-dependent manner (Upadhya et al., 2013). Srx1 is required for recycling of peroxiredoxin (Tsa1) and its deletion significantly attenuates the virulence of *C. neoformans* (Upadhya et al., 2013). Third, several kinases are regulated by the HOG pathway. These include Hrk1 (Hog1-regulated kinase), which is involved in osmoregulation (Kim et al., 2011), and Sch9, which is involved in thermotolerance and oxidative stress response, in *C. neoformans* (Kim et al., 2009). The *C. albicans* Hrk1 ortholog Rck2 was also shown to be induced by osmotic stress in a Hog1-dependent manner (Enjalbert et al., 2006). Fourth, Hog1-dependent transcription factors include Mbs1, which is involved in environmental stress response, ergosterol biosynthesis, membrane integrity, and virulence factor production in *C. neoformans* (Song et al., 2012). In particular, *MBS1* deletion attenuates virulence of *C. neoformans*. In *C. albicans*, the Sko1 transcription factor is transcriptionally regulated by Hog1 in response to osmotic stress and is also involved in the cell wall damage response (Enjalbert et al., 2006; Rauceo et al., 2008).

As hyperactivation of the HOG pathway is lethal to fungal cells, timely inactivation of the HOG pathway is critical for fungal cell growth. In *S. cerevisiae*, two tyrosine phosphatases (PTPs; Ptp2 and Ptp3) and three type 2C Ser/Thr phosphatases (PP2C; Ptc1, Ptc2, and Ptc3) are involved in negatively regulation of Hog1 (Maeda et al., 1994; Jacoby et al., 1997; Wurgler-Murphy et al., 1997). In *C. neoformans*, Ptp1 and Ptp2 were found to be transcriptionally regulated by the HOG pathway as negative feedback regulators (Lee et al., 2014). Particularly, Ptp2 plays a major role in regulating Hog1. Unexpectedly, some of the *ptp2*Δ mutant phenotypes, including increased susceptibility to osmotic, oxidative, and genotoxic stresses, are similar to those of the *hog1*Δ mutant, suggesting that coordinated, balanced regulation of the HOG pathway is critical for normal fungal cell physiology. Supporting this, *PTP2* deletion also attenuates the virulence of *C. neoformans* even more strongly than *HOG1* deletion (Lee et al., 2014). Notably, *PTP1* deletion exacerbates the virulence defect of the *ptp2*Δ mutant, suggesting that Ptp1 is also involved in the virulence of *C. neoformans* (Lee et al., 2014). In *C. albicans*, Ptp2 and Ptp3 are known to repress the basal Hog1 activity in response to Tor1 inhibition, which is required for hyphal maintenance (Su et al., 2013). However, the role of Ptp2 and Ptp3 in virulence of *C. albicans* has not been addressed. In contrast to PTPs, the role of any PP2Cs in Hog1 regulation remains unknown in *C. neoformans* and *C. albicans*.

### The Sho1/Msb2/Hkr1-Signaling Pathway

In addition to the multicomponent phosphorelay system, another upstream signaling branch has been reported to regulate the Ssk2-Pbs2-Hog1 MAPK module in *S. cerevisiae*: the Sho1/Msb2/Hkr1-signaling pathway. Msb2 and Hkr1 are two mucin-like transmembrane proteins and Sho1 is a membrane protein with the SH3 domain (Maeda et al., 1995; O’Rourke and Herskowitz, 2002; Tatebayashi et al., 2007). Msb2 and Hkr1 are bona fide osmosensors, which can physically interact with Sho1 through transmembrane domains and generate intracellular signaling through the cytoplasmic domain of Sho1. Sho1 also serves as an adaptor for recruiting Pbs2 and the Ste11/Ste50 complex (Maeda et al., 1995; Zarrinpar et al., 2004; Tatebayashi et al., 2006). In *C. neoformans*, Sho1 and Msb2 orthologs have been recently identified and functionally characterized (Kim et al., 2015; So et al., 2018). *SHO1* and *MSB2* deletions do not markedly affect Hog1 phosphorylation patterns in response to osmotic stress, suggesting that these proteins are not major Hog1 regulators in *C. neoformans*. Instead, Sho1 and Msb2 play redundant roles in phosphorylating the Cpk1 MAPK for cell wall biogenesis. However, deletion of *SHO1* or *MSB2* reduces Hog1 phosphorylation in the absence of Ssk1 (So et al., 2018), indicating that the Sho1 and Msb2 pathway may serve as a back-up signaling circuit for Hog1 activation when the major multicomponent phosphorelay system is shut down in *C. neoformans*. Notably, Msb2 and Sho1 are required for the early acute and later adaptation of *C. neoformans*, respectively, to the pulmonary environment during mammalian host infection (So et al., 2018).

Surprisingly, however, Ssk1, Sho1, and Msb2 are all dispensable for Hog1 phosphorylation in response to osmotic shock in *C. albicans* (Roman et al., 2009). In the triple *ssk1*Δ *sho1*Δ *msb2*Δ mutant, Hog1 can still be phosphorylated in response to osmotic shock, like the wild type strain in *C. albicans*, suggesting that another signaling branch may operate to activate Hog1. In *C. albicans*, the extracellular domain of Msb2 is cleaved and secreted as a highly glycosylated form during host infection and serves to protect fungal cells from antimicrobial peptides (Szafranski-Schneider et al., 2012). As the extracellular domain is not conserved between *C. neoformans* and *C. albicans* Msb2 orthologs, it remains unclear whether the extracellular domain of *C. neoformans* Msb2 is also cleaved and released during host infection.

## Receptors as Potential Drug Targets

Overall, sensing nutrient and stress signals is critical for fungal pathogens to adapt to the host environment to cause infection. As eukaryotic pathogens, fungi and the human host share much similarity in their cellular mechanisms, leading to very few targets for drug development that have both high potency and low toxicity. The cell surface sensors are in general more distinct compared to the intracellular signaling and metabolic pathways, and their accessible location is favorable for drug binding. Therefore, nutrient receptors could represent a potentially rich source of targets for antifungal drug development. Around 40% of all available drugs target GPCRs, making these receptors the most important drug target group. Transceptors are often important for fungal development and virulence, and hence could be an excellent drug target group as well. Indeed, receptors that sense different stress responses have been proposed as a drug targets.

Clinically, the fungal phosphorelay systems have drawn significant attention from a pharmaceutical standpoint because an equivalent system has not been discovered in mammals. In agriculture, some fungicides, for instance phenylpyrrole agents, primarily target the HHKs, which subsequently hyperactivates Hog1 and causes over-accumulation of intracellular glycerols, resulting in growth arrest (Kojima et al., 2006). Due to this reason, Ypd1, which is a central negative regulator of Hog1, has been considered as a prime target for antifungal drug development, particularly because any Ypd1 ortholog is not present in mammals. However, a recent report demonstrated that conditional repression of *YPD1* enhances the virulence of *C. albicans*, perhaps because reduced *YPD1* expression enhances stress resistance and filamentation (Day et al., 2017), which raising doubt as to whether Ypd1 is a viable target in *C. albicans*. In aggregate, increasing our understanding of nutrient sensing in fungal development and pathogenicity is critical and will enhance our ability to develop novel strategies to fight fungal diseases and antifungal drug resistance.

## Author Contributions

CX and JH designed the review. JR, Y-SB, BV, JH, and CX contributed to the writing of the manuscript.

## Conflict of Interest Statement

The authors declare that the research was conducted in the absence of any commercial or financial relationships that could be construed as a potential conflict of interest.
